# Implementation of Evidence-Based Practice Among Respiratory Therapists in Saudi Arabia: A Cross-Sectional Study

**DOI:** 10.3390/healthcare14030324

**Published:** 2026-01-27

**Authors:** Fahad H. Alahmadi, Ali M. Alasmari, Keir E. J. Philip, Ziyad Alshehri, Maher Aljohani, Majed K. Aljohani, Abdulrahman M. Hawsawi, Abdullah S. Alsulayyim, Rami A. Alyami, Yahya A. Alzahrani, Maher M. Alquaimi, Mohammed A. Almeshari, Batool Alnakhli, Nowaf Y. Alobaidi, Ahmed A. Alzahrani

**Affiliations:** 1 Respiratory Therapy Department, College of Medical Rehabilitation Sciences, Taibah University, Madinah 42353, Saudi Arabia; fhrahmadi@taibahu.edu.sa (F.H.A.); aasmri@taibahu.edu.sa (A.M.A.); zshahri@taibahu.edu.sa (Z.A.); ahawsawy@taibahu.edu.sa (A.M.H.); 2 National Heart and Lung Institute, Imperial College London, London SW7 2AZ, UK; k.philip@imperial.ac.uk; 3 Respiratory Care Services Line Office, Madinah Cluster, Madinah 42351, Saudi Arabia; 4 Respiratory Therapy Department, King Salman Bin Abdulaziz Medical City, Madinah 42210, Saudi Arabia; 5 Respiratory Therapy Program, Department of Nursing, College of Nursing and Health Sciences, Jazan University, Jazan 45142, Saudi Arabia; alsulayyim@jazanu.edu.sa (A.S.A.); ralyami@jazanu.edu.sa (R.A.A.); 6 Department of Respiratory Care, Imam Abdulrahman Bin Faisal University, Dammam 31441, Saudi Arabia; yaalzahrani@iau.edu.sa (Y.A.A.);; 7 Rehabilitation Health Sciences Department, College of Applied Medical Sciences, King Saud University, Riyadh 11543, Saudi Arabia; 8 Institute of Inflammation and Ageing, University of Birmingham, Birmingham B15 2TT, UK; 9 Head of Respiratory Therapy Department, Al-Salam Waqf Hospital, Al-Madinah Al-Munawwarah 35151, Saudi Arabia; 10 Respiratory Therapy Department, College of Applied Medical Sciences, King Saud Bin Abdulaziz University for Health Sciences, Alahsa 11481, Saudi Arabia; 11 King Abdullah International Medical Research Centre, Alahsa 11481, Saudi Arabia

**Keywords:** respiratory therapists, evidence-based practice, attitudes, knowledge, barriers, Saudi Arabia

## Abstract

**Highlights:**

**What are the main findings?**

- This study offers the first national-level insights into respiratory therapists’ (RTs’) attitudes, knowledge, and perceived barriers toward evidence-based practice (EBP) in Saudi Arabia;
- Findings reveal a generally positive disposition toward EBP; however, notable gaps exist in formal training, understanding of technical concepts, and institutional support.

**What are the implications of the main findings?**

- This study identifies key barriers to EBP integration, including limited access to resources, insufficient research skills, and an overreliance on personal experience;
- These findings underscore the urgent need for curriculum reform, targeted professional development, and policy initiatives to strengthen EBP in respiratory care.

**Abstract:**

**Background**: Evidence-based practice (EBP) has become a foundational component of modern healthcare globally. In Saudi Arabia, the understanding and application of EBP by respiratory therapists (RTs) remains underexplored. This study aimed to assess RTs’ behaviors, attitudes, awareness, knowledge, and barriers related to EBP. **Methods**: A previously validated online questionnaire was distributed to RTs across Saudi Arabia between February and July 2025. The survey collected sociodemographic data and included 14 items assessing behaviors, attitudes, awareness, knowledge, prior formal EBP training, and perceived barriers to EBP implementation. **Results**: A total of 301 RTs participated, with 290 completing the survey. Most participants (75.2%) held a bachelor’s degree. Overall, respondents demonstrated positive attitudes toward EBP, with more than 60% agreeing that understanding research methods is essential to respiratory therapy practice. The most frequently used resources for clinical decision-making were personal experience (67.3%), expert opinion (65.5%), and national or international guidelines (65.5%). Awareness of core EBP concepts was moderate; approximately 30% of participants reported a good understanding of terms such as “systematic review,” “quality of evidence,” and “risk of bias.” Several barriers to EBP implementation were identified, most commonly limited access to resources (25.2%), insufficient research knowledge and skills (23.8%), and lack of interest (21.0%). **Conclusions**: RTs in Saudi Arabia generally support EBP principles and use evidence-based resources in clinical decision-making. However, gaps in training, access to resources, and research competency limit full EBP implementation. Targeted strategies, including integrating mandatory EBP education, expanding professional development, and enhancing access to research resources, are recommended.

## 1. Background

Evidence-based practice (EBP) is a fundamental element of delivering high-quality care. EBP involves the integration of best current research evidence with clinical expertise and patient preferences to facilitate optimal clinical decision-making [1]. Since its formal introduction in the 1990s, EBP has evolved from a theoretical model into a practical framework that highlights patient safety, quality assurance, and healthcare sustainability [2,3]. One of the most frequently cited frameworks by Sackett and colleagues explains how EBP is about “integrating individual clinical expertise with the best external evidence” [4]. The EBP process has since been operationalized into five key phases: first, formulating a well-structured clinical question; second, finding the most relevant evidence; third, assessing how valid and useful that evidence is; fourth, integrating it into patient care; and finally, reviewing both the patient outcomes and how well the EBP process worked [4].

Over the past two decades, implementing evidence-informed healthcare has grown significantly, driven by increased attention to quality and patient-centered outcomes [5]. As a result, EBP has been incorporated into accreditation standards, academic curricula, and continuing professional development training across a range of clinical health professions [6].

Despite the dedicated institutional and professional support, the translation of EBP into clinical settings remains challenging. Studies across health professions, including nursing, physiotherapy, pharmacy, and medicine, have identified persistent barriers to EBP implementation, particularly in low- and middle-income countries [7,8]. Common challenges include limited time, poor access to evidence databases, insufficient institutional support, and a lack of confidence in interpreting research [9,10]. On the other hand, enablers of successful EBP integration include continuing education, mentoring programs, strong leadership support, and integration of EBP concepts into daily clinical routines [11,12]. Among allied health professionals, there is generally a favorable view of EBP, but many still express uncertainties about how to effectively apply research in clinical practice [13,14,15].

Respiratory therapists (RTs), as front-line healthcare professionals working across acute, critical, and long-term care settings, are uniquely positioned to both benefit from and contribute to EBP [16]. Yet, the international literature specific to RTs highlights notable gaps in self-efficacy, knowledge translation skills, and institutional support for EBP implementation [17,18]. For example, a study by Weng et al. (2014) [17] found that although RTs in Taiwan recognized the value of EBP, fewer than half consistently applied evidence in their clinical practice. Similar patterns have been observed in Canada and the United States, where RTs identified barriers such as limited training in research methodology and time constraints during clinical shifts [18,19].

In the context of Saudi Arabia, Vision 2030 presents a transformative national agenda aimed at enhancing healthcare services through innovation, workforce development, and the integration of evidence-based care. In alignment with this vision, various health sectors have introduced reforms to institutionalize EBP across healthcare disciplines [20]. Despite these initiatives, empirical studies continue to reveal a significant gap between EBP policy and its practical application. Research involving physiotherapists, pharmacists, and nurses in Saudi Arabia consistently reports low levels of EBP use in clinical decision-making. Commonly cited barriers include limited time, inadequate access to current evidence, and insufficient training in EBP principles [21,22,23].

Despite nationwide efforts to enhance the use of EBP across healthcare disciplines, its implementation in respiratory therapy remains relatively underexplored in Saudi Arabia. This gap is particularly significant given the vital role RTs play in managing complex cardiopulmonary conditions, especially in critical care settings, and the growing national emphasis on strengthening respiratory care education and workforce development [24]. Gaining insight into the current attitudes, knowledge, and barriers they might face regarding implementation of EBP is essential for improving clinical outcomes and for guiding the development of targeted educational programs and institutional support tools. Therefore, the aim of this study is to determine RTs’ behavior, attitudes, awareness, knowledge, and barriers in relation to EBP in Saudi Arabia.

## 2. Materials and Methods

### 2.1. Study Design

This study employed a cross-sectional national online survey distributed among RTs in Saudi Arabia between February and July 2025. Ethical approval was obtained from the College of Medical Rehabilitation Sciences Research Ethics Committee at Taibah University, Saudi Arabia (CMR-RT-2024-19/10-12-2024). All participants provided electronic informed consent prior to participation. This study adhered to, and is reported in accordance with, the Strengthening the Reporting of Observational Studies in Epidemiology (STROBE) checklist [25].

### 2.2. Population, Sampling, and Data Collection Process

The inclusion criteria were RTs (clinical or academic) holding a valid license in Saudi Arabia and actively practicing. Undergraduate RT students were excluded, as the expected outcomes of this study aim to inform post-professional educational development.

The target population comprised all RTs currently practicing in Saudi Arabia. According to the national workforce survey by Alotaibi (2015), the estimated reported number of RT staff is 1477 RTs [26]. Using a single-population proportion formula with a 95% confidence level (Z = 1.96), a 5% margin of error, and N = 1477, the required sample size was 305 RTs [27]. Additionally, to account for an anticipated 20% non-response rate, we increased the sample to 380 participants. A total of 301 responses were received. After screening for completeness and eligibility (e.g., currently practicing RTs in Saudi Arabia), 290 valid responses were included in the final analysis.

Participants were invited via a convenience sampling method and a snowballing technique [28]. Multiple digital platforms were employed to maximize reach and representation across Saudi Arabia. Distribution channels included social media platforms (X, WhatsApp groups) and direct emails sent through RT departmental managers in various healthcare institutions. Participation was voluntary, and all potential respondents were provided with an introductory statement explaining the purpose of this study, estimated time for completion, and a link to the electronic informed consent form. Only those who gave consent were able to proceed to the survey. To enhance response rates, reminder messages and social media posts were sent twice at two-week intervals via the same distribution channels. The survey was open for a total duration of approximately five months.

### 2.3. Data Collection Instrument

The survey instrument was originally developed and validated for use among physiotherapists in Saudi Arabia, and demonstrated acceptable reliability and content validity [21]. For the purposes of the present study, only minor contextual wording modifications were applied to ensure relevance to the respiratory therapy (RT) setting. These modifications were limited to adjustments in demographic items and the replacement of the term “physiotherapy” with “respiratory therapy,” without any changes to the questionnaire’s core content, structure, or scoring system.

The instrument comprised two main sections: The first gathered demographic and professional characteristics (e.g., age, gender, education, experience, work setting). The second section assessed EBP-related domains, including an awareness domain (14 items related to research terminology, measured using a five-point Likert scale ranging from 0 (never heard of it) to 4 (understand it completely)). The maximum possible score for this section was 56, with higher scores indicating a greater level of awareness regarding EBP. The knowledge domain comprised six items with response options of agree, disagree, or unsure. For all six items, only the disagree response was scored as correct, earning two points each, for a maximum score of twelve; higher scores reflected greater knowledge of EBP. The behavior domain assessed RTs’ use of EBP sources in daily clinical practice across six items using a frequency-based Likert scale. The attitude domain included four items rated on a five-point agreement scale, with higher scores representing more positive attitudes toward EBP implementation. Additional items evaluated formal EBP training (two questions) and perceived barriers (six statements ranked from 1 = least important barrier to 10 = most important barrier).

### 2.4. Data Analysis

Data analysis was carried out via SPSS^®^ 27.0 (IBM, Chicago, IL, USA). Continuous variables are presented as the means and standard deviations or medians and interquartile ranges (IQRs), whereas categorical variables are presented as frequencies and percentages.

## 3. Results

A total of 290 respondents were included in the final analysis of this study. The majority of participants were male (79.3%), and almost all were Saudi nationals (95.9%). Additionally, a bachelor’s respiratory therapy degree was the highest qualification for (75.2%) of participants, as shown in Table 1.

### 3.1. Behavior and Attitude to Evidence-Based Practice

Overall, participants reported behaviors aligning with, and positive attitudes towards, EBP. The majority reported frequent use of research reviews and articles (54.5%), personal experience (67.3%), expert opinion (65.5%), clinical guidelines (65.5%), and institutional protocols (57.2%) in their decision-making, as detailed in Table 2.

About 65.1% of respondents “strongly agreed” or “agreed” with the importance of understanding research methods and study designs in respiratory therapy practice, while 63.1% agreed/strongly agreed that regularly reading relevant research articles is essential for maintaining updated knowledge, as reported in Table 3.

### 3.2. Awareness and Knowledge of Evidence-Based Practice

Participants’ awareness of EBP terminology was variable. Approximately one-third reported a good understanding of general terms such as “EBP” and “EBP cycle”; however, a substantial proportion demonstrated only limited or no familiarity. Awareness declined further for technical terms like “forest plot” and “PICO,” whereas broader concepts such as “systematic review” and “quality of evidence” were relatively better understood, as outlined in Table 4.

Participants’ knowledge of EBP principles also demonstrated mixed levels of understanding. While over half (58.6%) correctly identified EBP as a systematic process for generating knowledge, misconceptions were common, including viewing it solely as a means to solve research problems or believing patient values and clinical experience are not essential components. Uncertainty about key principles, such as the time required for EBP implementation, further indicated limited understanding among many respondents, as presented in Table 5.

Moreover, the variability in EBP knowledge appeared to correspond with participants’ limited exposure to formal EBP training, as only 31.0% had received such training, while 69.0% had not, as indicated in Table 6.

### 3.3. Perceived Barriers to the Use of Evidence-Based Practice

**Figure 1:**
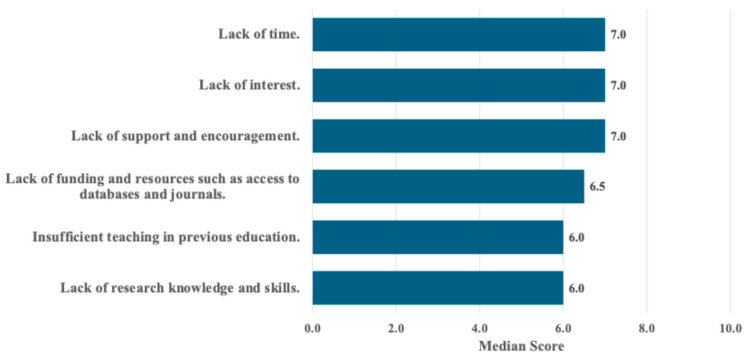
Barriers to the Use of Evidence-Based Practice reported by RTs in Saudi Arabia. Perceived barriers ranked from 1 (least important) to 10 (most important). Values are shown as the median score.

The main barriers to implementing EBP were lack of funding and resources (25.2%), limited research knowledge and skills (23.8%), and lack of interest (21.0%), and the Supplemental File shows this in more detail (Supplementary Materials Section). Structural limitations, educational gaps, and insufficient institutional support were also evident, indicating key obstacles to EBP adoption among RTs in Saudi Arabia, as displayed in Figure 1.

## 4. Discussion

This study explored behavior, attitudes, awareness, knowledge, and barriers regarding EBP among RTs in Saudi Arabia. RTs demonstrated positive attitudes toward EBP, predominantly agreeing with the importance of understanding research methodologies and reading the academic literature. However, there was less consistency in terms of awareness of technical EBP concepts, and the relatively low proportion of RTs with formal EBP training. This study identified areas in need of improvement relating to knowledge and awareness and identified barriers that may hinder the integration of EBP into clinical respiratory care.

### 4.1. Behavior and Attitudes to Evidence-Based Practice

Our results demonstrate that RTs were slightly more likely to rely on personal experiences rather than the literature and guidelines. These findings are in accordance with previous studies of a variety of professional healthcare groups [14,21,29]. In Saudi Arabia, Alshehri (2017) reported similar results and found that most physiotherapists (PTs) were more likely to make decisions based on personal experience acquired from clinical situations than recent, high-quality evidence [21]. In contrast, (89.5%) of Brazilian physiotherapists reported using research articles and guidelines as the first choice for their clinical decisions [30]. The tendency to rely on personal experience over clinical guidelines and research potentially reflects a systemic challenge and raises concerns about variability in care quality and alignment with best practices [16]. It is also possible that, due to hierarchies between different members of the multidisciplinary team, RTs may rely more on direction from physicians and have less autonomy regarding clinical decision-making.

Results also demonstrated that, in general, participants had a positive attitude towards the use of EBP, felt applying research in practice was important, and that interventions should be supported by evidence. This aligns with findings from Taiwan, where (86%) of RTs believed that EBP is important in patient care [17]. Likewise, Egyptian healthcare professionals expressed a positive attitude towards EBP and believed that EBP improves patient outcomes and decreases healthcare costs [31]. In the Saudi context, primary care physicians, nurses, and PTs agreed that there were benefits to the integration of EBP in decision-making for improving patient care [9,21,32].

Although participants demonstrated generally positive attitudes toward EBP, clinical decision-making remained largely influenced by personal experience and expert opinion. This attitude–behavior discrepancy has been widely described in implementation science and can be partially explained through frameworks such as the Capability–Opportunity–Motivation Behavior (COM-B) model and the Knowledge-to-Action (KTA) framework [33,34]. Within these models, positive attitudes reflect motivation; however, sustained behavior change also depends on sufficient capability (e.g., skills in critical appraisal and interpretation of statistical concepts) and opportunity (e.g., access to evidence, protected time, and supportive organizational cultures). While a positive attitude can show motivation, lasting behavior change also requires people to have the right skills—like the ability to critically assess and understand statistical ideas—as well as the opportunity to apply them. That includes having access to reliable evidence, dedicated time, and a supportive workplace environment.

### 4.2. Awareness and Knowledge of Evidence-Based Practice

Participants’ awareness of EBP-related terminology shows variation. While 24.8% of participants reported understanding terms such as “EBP”, “systematic reviews”, or “randomized controlled trial” very well, a substantially higher proportion (70%) had limited understanding of or had never heard the terms “EBP cycle or EBP steps”. Relatively few participants expressed high levels of confidence in relation to more technical terms such as “forest plot” and “relative risk”. This echoes findings from a study in Egypt that also found substantial variation in how familiar healthcare professionals were with using the terminology of EBP, which was mostly utilized by physicians, followed by pharmacists, with nurses the least likely to implement EBP [31]. Regarding knowledge, over half of the RTs reported familiarity with EBP terms, such as EBP definitions, aims, and agreement that all RTs’ interventions should be supported by EBP. These findings are inconsistent with previous studies. Reported research involving RTs from Taiwan and the United States has identified similar trends around positive attitudes but with low practical application due to limited knowledge of implementing EBP principles [17,18]. Likewise, studies among Saudi PTs, nurses, and pharmacists reported limited familiarity with EBP principles and underutilization of research in daily decision-making due to structural and educational barriers [21,22,23].

In this study, 69% of participants indicated they had not received formal training in EBP. This finding aligns with earlier research highlighting a general lack of structured EBP or research instruction in respiratory therapy education. For instance, Clark et al. found that although many respondents were familiar with basic EBP concepts, they struggled with more advanced elements, such as constructing PICO questions and interpreting statistical data [18]. Another national survey study in Taiwan reported that while 88% of RTs were aware of EBP, many lacked the practical skills necessary for its implementation, and only about half had ever applied it in clinical contexts [17]. Similarly, research in Saudi Arabia showed that 70.2% of PTs lacked formal EBP training [21]. These findings, including those from the current study, raise concerns about whether RT students in Saudi Arabia are receiving sufficient formal teaching in EBP, either through dedicated coursework or integrated curriculum components. Embedding EBP content into undergraduate and postgraduate curricula through journal clubs, critical appraisal assignments, and research projects is likely to lead to long-term improvements in EBP behaviors [6].

### 4.3. Barriers to EBP Implementation

Participants in this study reported three main barriers to implementing EBP, including limited access to research resources, insufficient research knowledge and skills, and lack of interest. These findings reflect the global literature on EBP challenges, where time constraints, resource shortages, and underdeveloped training infrastructure remain key limitations [8,11]. RTs often work in stressful clinical roles at the bedside in the intensive care unit [35]. For this reason, it is essential to create opportunities that allow RTs to engage in research, whether through providing time away from clinical duties, higher RT-to-patient ratios, or access to research training workshops.

### 4.4. Implications for Practice and Policy

The current study identifies multiple areas for improvement and perceived barriers to the implementation of EBP, each requiring targeted strategies for effective change. The limited understanding of technical EBP concepts, such as risk of bias, forest plots, and relative risk, suggests a need for targeted educational reform, including mandatory EBP modules within undergraduate respiratory therapy curricula that emphasize critical appraisal skills, research methodology, and applied statistics. Additionally, the observed reliance on personal experience and expert opinion in clinical decision-making underscores the importance of hospital support mechanisms, such as protected time within clinical schedules to allow RTs to appraise evidence and engage with current research. The reported lack of access to research resources further highlights the need for improved availability of online databases, clinical guideline repositories, and institutional subscriptions. Finally, clear pro-EBP position statements and advocacy from national regulatory bodies and professional societies, embedded within accreditation standards and clinical guidelines, would be useful. These improvement interventions would not only improve professional competency but also enhance patient care, aligning respiratory therapy with the strategic goals of Saudi Arabia’s Vision 2030, which emphasizes innovation, healthcare quality, and data-driven decision-making.

### 4.5. Strengths and Limitations

This study has several strengths. It offers a national-level assessment of EBP engagement among respiratory therapists in Saudi Arabia, includes a large and diverse sample, and utilizes a previously validated questionnaire. However, some limitations must be acknowledged. First, the cross-sectional design captures data at a single point in time, preventing the assessment of changes over time. Second, self-reported data are subject to recall and social desirability bias, although the anonymous nature of participation likely mitigated some of this risk. Importantly, the use of convenience and snowball sampling may have introduced selection bias, limiting the generalizability of the findings. This approach may have led to the overrepresentation of RTs who are already interested in research or EBP, thereby inflating the reported levels of positive attitudes and engagement. As a result, the findings may not fully reflect the views of the broader RT population in Saudi Arabia, particularly those with limited exposure to EBP. In addition, the questionnaire was not re-validated for the respiratory therapy population, as only minor contextual wording changes were made without modifying the core items, constructs, or scoring. Future research may confirm the instrument’s measurement properties specifically among respiratory therapists.

Future research should aim to use probabilistic sampling methods and larger, more representative samples to validate and expand upon these findings. Additionally, studies investigating the relationship between perspectives of EBP and its implementation could be useful. Assessing the relationship between differing training programs and subsequent perspectives and practice could provide insights to design future training programs.

## 5. Conclusions

This study found that although many RTs in Saudi Arabia have generally positive attitudes regarding EBP, there are substantial gaps in knowledge, training, and practical application. The most frequently reported barriers included limited access to reliable resources such as the literature databases, a lack of appropriate research skills, and insufficient institutional support. Targeted strategies, such as embedding mandatory EBP training in curricula, strengthening ongoing professional development, and facilitating better access to research tools, are recommended.

## Figures and Tables

**Table 1:** Demographics of study participants.

Variable	Demographics	Count (%)
Gender	Female	60 (20.7%)
	Male	230 (79.3%)
Age groups (year)	18–25	95 (32.8%)
	26–30	114 (39.3%)
	31–35	40 (13.8%)
	36–40	20 (6.90%)
	41 or more	21 (7.20%)
Nationality	Non-Saudi	12 (4.10%)
	Saudi	278 (95.9%)
Education	Diploma	17 (5.90%)
	Bachelor’s degree	218 (75.2%)
	Master’s degree	42 (14.5%)
	PhD	13 (4.50%)
Occupation	Clinician (Respiratory Therapist)	260 (89.6%)
	Academic Respiratory Therapist staff	11 (3.80%)
	Both clinician and academic staff	19 (6.60%)
Work setting	Ministry of Health	181 (62.4%)
	University/Academic hospital	33 (11.4%)
	Other sectors such as National Guard, ARAMCO, Armed Forces, Security Forces, and Royal Commission	31 (10.7%)
	Private health sector	45 (15.5%)
Years of Work	<1 year	29 (10.0%)
	1–4 years	133 (45.9%)
	5–10 years	81 (27.9%)
	>10 years	47 (16.2%)
SCFHS classification	Consultant	6 (2.10%)
	Senior Specialist	41 (14.1%)
	Specialist	231 (79.7%)
	Technician	12 (4.10%)

**Table 2:** Participants’ behavior toward the use of research and other sources when making daily clinical decisions. Values are shown as N (%).

Sources	Always	Often	Sometimes	Rarely	Never
My personal experience	111 (38.3%)	84 (29.0%)	86 (29.7%)	8 (2.8%)	1 (0.3%)
My colleagues’ opinions	51 (17.6%)	90 (31.0%)	123 (42.4%)	23 (7.9%)	3 (1.0%)
My supervisor’s or expert opinions	94 (32.4%)	96 (33.1%)	85 (29.3%)	13 (4.5%)	2 (0.7%)
Internet	82 (28.3%)	54 (18.6%)	103 (35.5%)	41 (14.1%)	10 (3.4%)
Books	82 (28.3%)	74 (25.5%)	92 (31.7%)	39 (13.4%)	3 (1.0%)
Research reviews and articles	96 (33.1%)	62 (21.4%)	81 (27.9%)	44 (15.2%)	7 (2.4%)
Workplace protocol only	88 (30.3%)	78 (26.9%)	94 (32.4%)	24 (8.3%)	6 (2.1%)
International/national guidelines	108 (37.2%)	82 (28.3%)	61 (21.0%)	32 (11.0%)	7 (2.4%)

**Table 3:** Participants’ attitudes toward evidence-based practice implementation. Values are shown as N (%).

Statement	Strongly Agree	Agree	Neutral	Disagree	Strongly Disagree
Understanding of research methods and research designs is important in respiratory therapy practice.	72 (24.8%)	88 (30.3%)	80 (27.6%)	24 (8.3%)	26 (9.0%)
Research theory and methodology should be included in the respiratory therapy curriculum.	76 (26.2%)	101 (34.8%)	71 (24.5%)	22 (7.6%)	20 (6.9%)
Respiratory therapists need to read relevant articles regularly to update their knowledge.	99 (34.1%)	84 (29.0%)	64 (22.1%)	19 (6.6%)	24 (8.3%)
Respiratory therapists should apply treatments that are supported by evidence.	113 (39.0%)	63 (21.7%)	79 (27.2%)	14 (4.8%)	21 (7.2%)

**Table 4:** Participants’ awareness of EBP terminology. Values are shown as N (%).

Terminology	Understand Very Well	Understand Completely and Could Explain to Others	Understand a Little	Have Heard It but Do Not Understand	Never Heard It
EBP as a term	72 (24.8%)	37 (12.8%)	104 (35.9%)	32 (11.0%)	45 (15.5%)
EBP cycle/steps	71 (24.5%)	14 (4.8%)	93 (32.1%)	55 (19.0%)	57 (19.7%)
Quality of evidence	91 (31.4%)	47 (16.2%)	109 (37.6%)	24 (8.3%)	19 (6.6%)
Systematic review	99 (34.1%)	60 (20.7%)	86 (29.7%)	28 (9.7%)	17 (5.9%)
PICO	70 (24.1%)	30 (10.3%)	88 (30.3%)	49 (16.9%)	53 (18.3%)
Critical appraisal	81 (27.9%)	35 (12.1%)	98 (33.8%)	35 (12.1%)	41 (14.1%)
Forest plot	41 (14.1%)	23 (7.9%)	94 (32.4%)	58 (20.0%)	74 (25.5%)
Relative risk	72 (24.8%)	38 (13.1%)	101 (34.8%)	49 (16.9%)	30 (10.3%)
Likelihood ratio	71 (24.5%)	34 (11.7%)	104 (35.9%)	46 (15.9%)	35 (12.1%)
Confidence interval	67 (23.1%)	35 (12.1%)	104 (35.9%)	53 (18.3%)	31 (10.7%)
Effect size	84 (29.0%)	31 (10.7%)	93 (32.1%)	46 (15.9%)	36 (12.4%)
Risk of bias	80 (27.6%)	53 (18.3%)	87 (30.0%)	43 (14.8%)	27 (9.3%)
Healthcare databases	63 (21.7%)	42 (14.5%)	114 (39.3%)	41 (14.1%)	30 (10.3%)

**Table 5:** Participants’ knowledge of evidence-based practice principles. Values are shown as N (%).

Statements	Agree	Unsure	Disagree
EBP is a process of systematic investigation to generate knowledge and test theories.	170 (58.6%)	88 (30.3%)	32 (11.0%)
The main aim of EBP is to identify the causes of research problems and how to solve them.	136 (46.9%)	94 (32.4%)	60 (20.7%)
Respiratory therapy interventions are mostly supported by EBP.	158 (54.5%)	105 (36.2%)	27 (9.3%)
Patient’s values and preferences are not one of the main requirements of EBP.	77 (26.6%)	126 (43.4%)	87 (30.0%)
EBP does not take into consideration the clinical experience of respiratory therapists.	81 (27.9%)	123 (42.4%)	86 (29.7%)
EBP requires a short period of time to search for, evaluate, and integrate evidence into practice.	76 (26.2%)	119 (41.0%)	95 (32.8%)

**Table 6:** Participants’ responses regarding EBP training. Values are shown as N (%).

Items	Answers	N (%)
Have you formally undertaken any training in EBP?	No	200 (69.0%)
	Yes	90 (31.0%)
If yes, what type of training course have you been involved in?	EBP course as part of university education (more than 20 h)	19 (6.6%)
	Comprehensive course (11 to 20 h)	16 (5.5%)
	Short course (3 to 10 h)	30 (10.3%)
	One lecture (1 to 2 h)	25 (8.6%)

## Data Availability

The dataset used and analyzed during the current study is available from the corresponding author upon request. The data are not publicly deposited; however, full access can be granted to the journal and reviewers.

## Supplementary Materials

The following supporting information can be downloaded at: https://www.mdpi.com/article/10.3390/healthcare14030324/s1, Table S1: Barriers to the Use of Evidence-Based Practice. Values are shown as N (%).
