# Supplementary material for: Implementation of Evidence-Based Practice Among Respiratory Therapists in Saudi Arabia: A Cross-Sectional Study
Source: Healthcare (Basel). 2026 Jan 27;14(3):324. doi: 10.3390/healthcare14030324 (PMC12897039; doi:10.3390/healthcare14030324)
Supplement: Supplementary file 1 [file healthcare-14-00324-s001.zip › healthcare-4077864-supplementary.pdf]

Supplementary Data

Table S1. Barriers to the Use of Evidence-Based Practice. Values are shown as N (%).

Barrier	**10**	**9**	**8**	**7**	**6**	**5**	**4**	**3**	**2**	**1**
Lack of research knowledge and skills	69 (23.8%)	21 (7.2%)	28 (9.7%)	21 (7.2%)	25 (8.6%)	56 (19.3%)	13 (4.5%)	20 (6.9%)	22 (7.6%)	15 (5.2%)
Lack of support and encouragement	58 (20.0%)	37 (12.8%)	31 (10.7%)	24 (8.3%)	31 (10.7%)	36 (12.4%)	16 (5.5%)	19 (6.6%)	20 (6.9%)	18 (6.2%)
Insufficient teaching in previous education	54 (18.6%)	28 (9.7%)	38 (13.1%)	20 (6.9%)	26 (9.0%)	49 (16.9%)	16 (5.5%)	24 (8.3%)	22 (7.6%)	13 (4.5%)
Lack of time	49 (16.9%)	30 (10.3%)	39 (13.4%)	32 (11.0%)	22 (7.6%)	42 (14.5%)	14 (4.8%)	15 (5.2%)	23 (7.9%)	24 (8.3%)
Lack of interest	61 (21.0%)	35 (12.1%)	29 (10.0%)	21 (7.2%)	26 (9.0%)	40 (13.8%)	18 (6.2%)	19 (6.6%)	19 (6.6%)	22 (7.6%)
Lack of funding and resources	73 (25.2%)	40 (13.8%)	16 (5.5%)	16 (5.5%)	28 (9.7%)	41 (14.1%)	13 (4.5%)	21 (7.2%)	22 (7.6%)	20 (6.9%)
